# Insomnia Severity in Psychiatric Outpatients: Real-World Insomnia Severity Index Data from an Italian Community Mental Health Center

**DOI:** 10.3390/brainsci16060617

**Published:** 2026-06-09

**Authors:** Vassilis Martiadis, Enrico Pessina, Azzurra Martini, Marco Marzolla, Chiara Bergesio, Francesca Barbaro, Alex Cavallo, Fabiola Raffone, Carlo Ignazio Cattaneo

**Affiliations:** 1 Department of Mental Health, Asl Napoli 1 Centro, 80125 Naples, Italy; 2 Department of Mental Health, Asl Cuneo 2, 12042 Bra, Italy; 3 Casa di Cura San Michele, 12042 Bra, Italy; 4 Department of Mental Health, Asl Biella, 13900 Biella, Italy; 5 Department of Psychiatry, University of Campania “L. Vanvitelli”, 80145 Naples, Italy

**Keywords:** insomnia, Insomnia Severity Index, psychiatric outpatients, depression, ADHD, community mental health, real-world data

## Abstract

Background: Insomnia is common among people with mental health conditions and can exacerbate symptoms, impair functioning and negatively impact treatment outcomes. Community mental health services require practical data to quantify the burden of insomnia in routine care and to identify groups at a higher risk of experiencing clinically significant insomnia. Methods: We conducted a retrospective analysis of anonymized routinely collected clinical data from adult psychiatric outpatients attending the Community Mental Health Center in Bra (Department of Mental Health, Asl Cuneo 2, Italy). Consecutive patients were included over a three-month period (1 September to 30 November 2025). Insomnia severity was assessed using the Insomnia Severity Index (ISI). Diagnoses were established by psychiatrists using the Structured Clinical Interview for DSM-5 (SCID-5). Results: The sample included 506 patients (mean age: 45.1 ± 16.7 years; 265 women, 52.4%). The mean ISI total score was 12.18 ± 6.99. Clinically significant insomnia (ISI ≥ 15) was present in 205 out of 506 patients (40.5%), while severe insomnia (ISI ≥ 22) was present in 55 out of 506 patients (10.9%). The ISI score differed across diagnostic groups (ANOVA, F(8, 497) = 2.82, *p* = 0.0046, η^2^ = 0.043). Post hoc comparisons revealed higher ISI scores in patients with depressive disorders than in those with anxiety disorders (Tukey, *p* = 0.0056). In a multivariable logistic regression model (outcome: ISI score of at least 15), adjusted for age, sex, education and the complexity of concurrent psychotropic medication (number of medication classes), depressive disorders were associated with clinically significant insomnia (OR: 1.99; 95% CI: 1.07–3.73). Attention deficit hyperactivity disorder (ADHD) also showed higher odds (OR: 3.64; 95% CI: 1.26–10.55). Medication complexity was also associated with an ISI score of at least 15 (OR: 1.43 per additional class; 95% CI: 1.16–1.77). In a sensitivity model additionally adjusting for benzodiazepine prescription (yes/no), benzodiazepine prescription was associated with ISI ≥ 15 (OR 1.82; 95% CI 1.13–2.95), while the estimate for medication complexity was attenuated using this association (OR 1.17; 95% CI 0.90–1.53). The eating disorders group was excluded from multivariable models due to the very small sample size (*n* = 4). Conclusions: Clinically significant insomnia was prevalent among this sample of psychiatric outpatients, with modest differences in insomnia severity across diagnostic groups. Sensitivity analyses suggested that the signal of medication complexity may be partly accounted for by benzodiazepine prescribing, supporting the cautious interpretation of medication-related correlates in routine cross-sectional data. These findings support routine insomnia screening in psychiatric outpatient care, while prospective studies are needed to clarify directionality and clinical implications.

## 1. Plain Language Summary

Sleep problems are common in psychiatric care, but they are not always assessed systematically. We analyzed anonymized data collected during routine practice at an Italian community mental health center over a three-month period in 2025. Patients completed a short questionnaire (the Insomnia Severity Index), which measures the severity of insomnia. Approximately 41% of patients had scores indicating clinically significant insomnia. There was a modest difference in insomnia severity across diagnostic groups, with depressive disorders being associated with a higher likelihood of clinically significant insomnia. Benzodiazepine prescriptions were common and were associated with clinically significant insomnia. Patients who were prescribed a greater variety of psychotropic medications were also more likely to report clinically significant insomnia. These findings support the routine screening of patients for insomnia in community psychiatric services to help identify those who may require a more detailed, sleep-focused assessment and access to evidence-based interventions.

## 2. Introduction

Insomnia disorder is common and disabling. In psychiatric care, it often presents as a persistent problem that exacerbates distress, impairs daytime functioning and complicates treatment planning. Current clinical guidance emphasizes that insomnia should be assessed and treated as a disorder in its own right, even when it occurs alongside mental health conditions, and recommends cognitive behavioral therapy for insomnia (CBT-I) as the primary treatment approach [1,2,3]. Historically, sleep disturbance in psychiatry has mainly been considered a symptom that reflects the progression of the primary disorder. In contrast, transdiagnostic models describe insomnia as an active process that contributes to the onset and persistence of multiple psychiatric conditions through hyperarousal, maladaptive sleep behaviors and impaired emotion regulation. This creates self-reinforcing cycles between poor sleep and psychopathology [4,5]. Longitudinal evidence supports this perspective. Insomnia symptoms predict the onset of depression, and meta-analytic data indicate that sleep disturbances are associated with the first occurrence of major mental health conditions during adolescence and early adulthood. Population studies have also linked insomnia to the incidence, recurrence and persistence of common mental disorders, suggesting that persistent sleep problems may act as a maintaining factor across diagnostic boundaries [6,7,8]. Despite an increasing body of evidence, including meta-analyses showing that CBT-I is effective in patients with comorbid mental disorders [9], and randomized trials demonstrating that improving sleep can reduce mental health symptoms [10,11], systematic insomnia assessment is not consistently embedded in routine psychiatric outpatient workflows. Service-level studies continue to report high rates of clinically significant insomnia alongside gaps in access to structured insomnia care. This underscores the need for pragmatic, real-world data to inform feasible pathways within community services [9,10,12,13].

In addition to the primary psychiatric diagnosis, the complexity of the pharmacological treatment of the underlying condition may contribute to the burden of insomnia in routine care. Psychotropic regimens often include combinations of medication classes prescribed not only for core symptoms, but also for sleep disturbances, agitation and distress. Therefore, a pragmatic marker such as the number of concurrent psychotropic medication classes prescribed can capture the overall clinical complexity and context of prescribing, although it does not allow for the attribution of causality to specific drugs. As routine medication information was consistently available at the level of medication class, we defined medication complexity as the number of concurrent psychotropic medication classes, rather than medication-level polypharmacy. We conceptualized this variable primarily as an indicator of overall clinical complexity (including severity of illness/treatment resistance and prescribing context) rather than as a sleep-specific intervention or a direct pharmacological driver of insomnia. Against this background, we used routinely collected clinical data from a community mental health center to: (i) describe the distribution and severity of insomnia symptoms in a consecutive real-world outpatient cohort, (ii) compare insomnia severity across broad diagnostic groups, and (iii) examine whether clinically significant insomnia was associated with diagnostic category and selected clinical features, including medication complexity.

## 3. Materials and Methods

### 3.1. Study Design, Reporting, and Setting

We conducted a retrospective observational study based on anonymized routinely collected clinical data. The study was performed at the Community Mental Health Center (CMHC) in Bra, Department of Mental Health, ASL Cuneo 2, Italy. We followed STROBE (Strengthening the Reporting of Observational Studies in Epidemiology) guidelines for reporting observational studies.

### 3.2. Participants and Recruitment Window

All adult outpatients (aged ≥ 18 years) attending the CMHC for either an initial or follow-up appointment between 1 September and 30 November 2025 were eligible. Sampling was consecutive within the predefined window. Here, ‘consecutive’ refers to patient identification (i.e., all eligible outpatients attending within the specified timeframe). The ISI was administered routinely to all outpatients attending the service during the recruitment window, regardless of diagnosis or symptom severity. However, as this was a retrospective analysis of routinely collected data, reasons for non-completion or refusal were not systematically recorded. Each patient contributed a single observation; if more than one assessment occurred during the recruitment period, only the first ISI assessment was included to avoid duplication of data within individuals. The single-observation retention rule was only applied to avoid duplicate observations from the same individual. Patients were included if a primary psychiatric diagnosis and a complete ISI assessment were available. An STROBE-style flow diagram summarizing patient selection and deduplication is provided in Figure S1.

### 3.3. Clinical Diagnoses

Primary psychiatric diagnoses were established by psychiatrists using the Structured Clinical Interview for DSM-5 (SCID-5), either at the initial assessment or during a prior diagnostic work-up in follow-up patients [14]. For initial assessments within the recruitment period, the SCID-5 diagnostic interview was conducted as part of standard intake procedures. For follow-up visits, diagnoses established using the SCID-5 during the initial diagnostic assessment were confirmed during routine clinical review. For the purposes of the analyses, diagnoses were grouped into the following broad categories: anxiety disorders, depressive disorders, bipolar disorders, psychotic disorders, personality disorders, obsessive–compulsive disorder, adjustment disorder, ADHD and eating disorders. Within these broad categories, the most frequent primary diagnoses were: anxiety disorders (generalized anxiety disorder, *n* = 38; panic disorder, *n* = 37; social anxiety/phobia, *n* = 4; other anxiety diagnoses, *n* = 5), depressive disorders (major depressive disorder, *n* = 120), bipolar disorders (bipolar II disorder, *n* = 81; bipolar I disorder, *n* = 44), psychotic disorders (mainly schizophrenia spectrum disorders, *n* = 27; schizoaffective disorder, *n* = 9; delusional disorder, *n* = 7; psychosis NOS/other, *n* = 7; other diagnoses recorded within the psychotic disorders category, *n* = 2), personality disorders (predominantly borderline personality disorder, *n* = 40; other/unspecified personality disorders, *n* = 2), obsessive–compulsive and related disorders (OCD, *n* = 29; hoarding disorder, *n* = 2), adjustment disorders (*n* = 29), ADHD (*n* = 19), and eating disorders (anorexia nervosa, *n* = 1; bulimia nervosa, *n* = 2; binge-eating disorder, *n* = 1). Psychiatric comorbidity was defined as having at least one additional current psychiatric diagnosis, in addition to the primary diagnosis, as recorded in routine clinical documentation.

### 3.4. Insomnia Assessment

Insomnia symptoms were assessed using the Insomnia Severity Index (ISI), a self-report measure consisting of seven items, each rated on a scale of 0–4 (total score range: 0–28), referring to the previous two weeks. Standard cut-offs were used to categorize clinical severity (0–7: none; 8–14: subthreshold; 15–21: moderate; 22–28: severe) and to define clinically significant insomnia (ISI ≥ 15) [15,16,17]. All ISI questionnaires included in the analytical dataset were completed in full. Therefore, there were no partially completed questionnaires and no missing values for any of the items.

### 3.5. Other Variables and Medication Coding

Demographic variables (age, sex and years of education) and the presence of psychiatric comorbidity were extracted from routine clinical records. Medication variables were coded at the class level (antidepressants, antipsychotics, benzodiazepines, antiepileptics/mood stabilizers, lithium, Z-drugs, and other psychotropics). Newer hypnotics, such as dual orexin receptor antagonists and melatonin receptor agonists, were not routinely prescribed at our center during the study period due to limited reimbursement and high costs in the public setting. Therefore, they were not included in the routine medication dataset. Medication complexity was operationalized as the number of concurrent psychotropic medication classes (range 0–5). Psychotropic polypharmacy (≥2 psychotropic medications) was recorded descriptively. Medication data were available at the class level only, without information on dose, timing, duration, or prescribing indication.

### 3.6. Data Extraction, De-Identification, and Quality Checks

Data were exported from the clinical information system after the recruitment period using a visit-date query. Prior to analysis, the dataset was de-identified by removing direct identifiers and date fields, while retaining only anonymized study IDs and non-identifying variables. Data quality checks included range checks for ISI items (0–4), verification of total score consistency and confirmation of one record per patient.

### 3.7. Statistical Analysis

Continuous variables are reported as the mean ± standard deviation, while categorical variables are reported as counts and percentages. Differences in the total ISI score across diagnostic groups were tested using one-way ANOVA with Tukey’s post hoc tests, and the effect size was reported as eta-squared (η^2^). A Kruskal–Wallis test was performed as a non-parametric sensitivity analysis. Associations between diagnostic group and ISI severity categories were examined using chi-squared tests, with Cramer’s V reported as the effect size. The analyses were pre-specified as primary (total ISI score across diagnostic groups and prevalence of ISI ≥ 15), secondary (nocturnal and daytime impact domains) and exploratory (item-level analyses). For exploratory item-level comparisons, *p*-values were adjusted using the Benjamini–Hochberg false discovery rate (FDR) procedure. To explore the correlates of clinically significant insomnia (ISI ≥ 15), multivariable logistic regression models were fitted, including the following variables: diagnostic group (reference: anxiety disorders); age; sex; years of education; and medication complexity (number of concurrent psychotropic medication classes). Anxiety disorders were chosen as the reference category because they were a clinically relevant group to compare with in this outpatient setting, and there was an adequate sample size. Due to the very small size of the eating disorders group (*n* = 4), this group was excluded from the multivariable regression models to prevent unstable estimates. Predicted probabilities were obtained from the fitted model with the covariates held at their sample means. As a sensitivity analysis, an ordinal logistic regression model was fitted with ISI severity categories as an ordered outcome, using the same covariates. As an additional sensitivity analysis, we refitted the multivariable logistic regression model, this time including benzodiazepine prescription (yes/no) as a binary covariate. This was done given the high prevalence of benzodiazepine prescriptions and their potential to distort estimates of insomnia severity in routine psychiatric care. Psychiatric comorbidity was examined descriptively, but it was not included as an additional covariate in the primary multivariable models to avoid redundancy with the primary diagnostic group and to preserve model stability. In an exploratory check, adding comorbidity did not materially change the pattern of estimates. All questionnaires were complete, so there were no missing data for ISI items. Analyses were performed using SPSS Software (version 22).

### 3.8. Ethics and Consent

This study involved a retrospective observational analysis of routinely collected clinical data. Identifiable information remained within the clinical record, and the analytic dataset was de-identified prior to statistical analysis, containing no direct identifiers. In accordance with local regulations and institutional requirements for retrospective analyses of routine clinical data involving no intervention or impact on clinical care, approval from the Ethics Committee/IRB was not required. Written informed consent for clinical assessment and for the use of de-identified routine clinical data for research and teaching purposes was obtained from all participants prior to the inclusion of their data. This manuscript does not include any patient-identifiable information.

## 4. Results

### 4.1. Sample Characteristics

The analytic sample included 506 patients. The mean age was 45.1 years (SD 16.7) and 265 participants were female (52.4%). At the time of assessment, most patients (463/506, 91.5%) were receiving psychotropic medication. The mean medication complexity was 1.86 ± 1.03 classes. Full sample characteristics and medication class prevalence are reported in Table 1.

### 4.2. Distribution of Insomnia Severity

**Figure 1:**
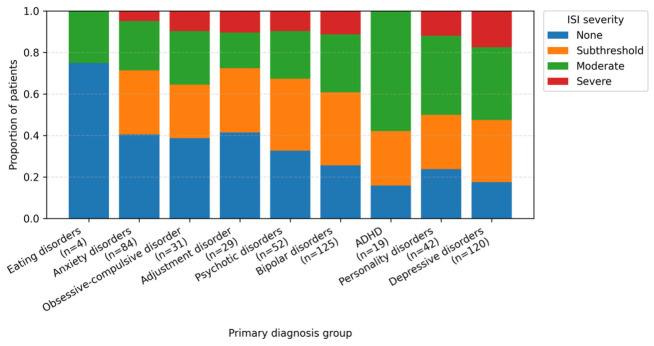
Distribution of ISI severity categories by diagnostic group. Bars represent within-group proportions of none (0–7), subthreshold (8–14), moderate (15–21), and severe (22–28) insomnia. Abbreviations: ADHD, attention-deficit/hyperactivity disorder; ISI, Insomnia Severity Index; OCD, obsessive–compulsive disorder.

The mean ISI total score was 12.18 ± 6.99. Clinically significant insomnia (ISI ≥ 15) was present in 205/506 patients (40.5%), while severe insomnia (ISI ≥ 22) was present in 55/506 patients (10.9%). The overall distribution of ISI severity categories is reported in Table 1, and the distribution by diagnostic group is shown in Figure 1.

### 4.3. Insomnia Severity Across Diagnostic Groups

**Figure 2:**
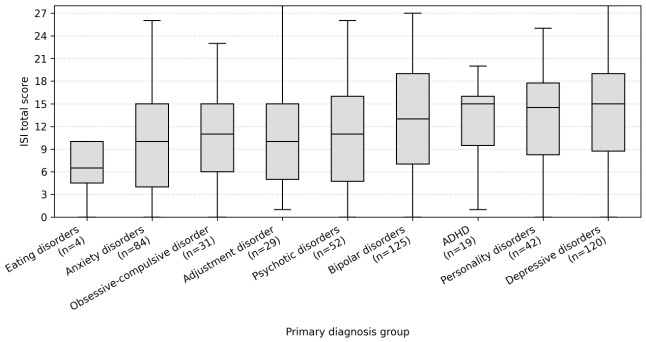
ISI total score by diagnostic group. Boxplots show the median and interquartile range (IQR); whiskers represent the central distribution (outliers not shown). Abbreviations: ADHD, attention-deficit/hyperactivity disorder; ISI, Insomnia Severity Index; IQR, interquartile range; OCD, obsessive–compulsive disorder.

The diagnostic group was associated with ISI severity categories (χ^2^ = 41.44, df = 24, *p* = 0.0149; Cramer’s V = 0.165). ISI total scores differed across diagnostic groups (ANOVA: F(8, 497) = 2.82, *p* = 0.0046, η^2^ = 0.043). Tukey’s post hoc test revealed a significant difference between depressive and anxiety disorders (mean difference: 3.7 points; *p* = 0.0056; Hedges’ g = 0.53), with depressive disorders exhibiting higher ISI scores (see Table 2 and Figure 2). The Kruskal–Wallis test yielded similar results (H = 22.77, *p* = 0.0037).

### 4.4. Domain and Item-Level Patterns

**Figure 3:**
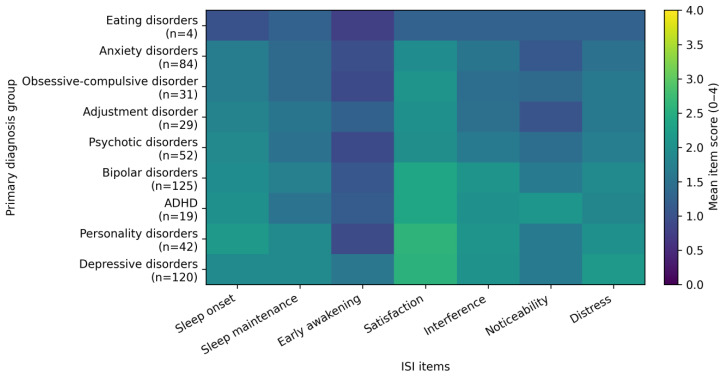
Heatmap of mean ISI item scores (0–4) by diagnostic group. ISI items: 1a sleep onset; 1b sleep maintenance; 1c early awakening; 2 sleep satisfaction; 3 interference with daytime functioning; 4 noticeability of impairment; 5 distress. Abbreviations: ADHD, attention-deficit/hyperactivity disorder; ISI, Insomnia Severity Index; OCD, obsessive–compulsive disorder.

Group differences were observed for nocturnal symptoms (sleep onset, maintenance and early awakening; F(8, 497) = 2.20, *p* = 0.0260) and perceived daytime impact/distress (items 2–5; F(8, 497) = 3.07, *p* = 0.0022). Figure 3 shows mean item profiles by diagnostic group. Following Benjamini–Hochberg FDR correction, significant differences between groups were retained for early morning awakening (item 1c) and the perceived impact/distress items (items 2–5). Sleep-onset and sleep-maintenance items, however, did not remain significant. This pattern suggests that differences between groups were driven more by early morning awakening and the perceived impact and distress related to daytime functioning than by sleep-onset or sleep-maintenance issues alone.

### 4.5. Clinically Significant Insomnia: Multivariable Models

**Figure 4:**
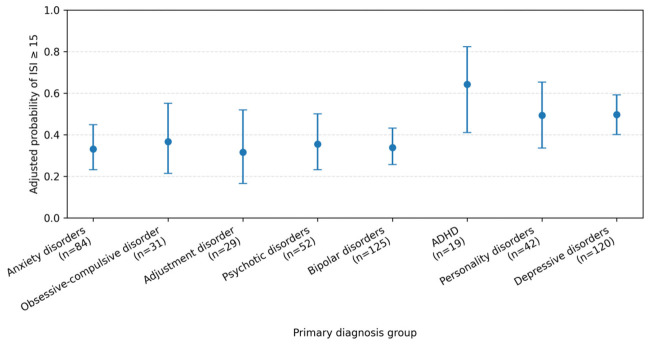
Adjusted predicted probability of clinically significant insomnia (ISI ≥ 15) by diagnostic group, as modeled using multivariable logistic regression. The points represent adjusted probabilities and the error bars indicate 95% CI. The model was adjusted for age, sex, years of education, and medication complexity (i.e., the number of concurrent psychotropic medication classes). The eating disorders group was excluded from the multivariable model due to the very small sample size (*n* = 4). Abbreviations: CI, confidence interval; ISI, Insomnia Severity Index.

In multivariable logistic regression (outcome: ISI ≥ 15), adjusted for age, sex, years of education, and medication complexity, ADHD and depressive disorders were independently associated with higher odds of clinically significant insomnia compared with anxiety disorders (Table 3; Figure 4). Medication complexity was also independently associated with ISI ≥ 15 (Table 3). The eating disorders group was excluded from multivariable models due to the very small sample size (*n* = 4). Internal consistency of the ISI was high in this sample (Cronbach’s α = 0.924; Table S1). The diagnostic group was associated with ISI severity categories (see Table S2). The distribution of medication classes by diagnostic group (including benzodiazepines and Z-drugs) is reported in Table S3. In an ordinal logistic sensitivity model using ordered ISI severity categories and the same covariates as the primary model, ADHD, depressive disorders, and medication complexity remained significant correlates (see Table S4). Multiple medication classes were not included as covariates in the primary model simultaneously because information on indication, dose and duration was unavailable, and because medication classes are correlated with the overall medication complexity measure. However, given the potential confounding role of benzodiazepines, we performed a sensitivity model additionally adjusting for benzodiazepine prescription (yes/no) showing that benzodiazepine prescription was associated with ISI ≥ 15 (OR 1.82; 95% CI 1.13–2.95; *p* = 0.015), whereas the estimate for medication complexity was attenuated (OR 1.17; 95% CI 0.90–1.53; *p* = 0.233) (Table S5).

## 5. Discussion

In this consecutive real-world cohort of psychiatric outpatients assessed in routine care at an Italian community mental health center, clinically significant insomnia was prevalent: approximately 40% of patients scored in the moderate-to-severe range [1,13]. Although insomnia burden was present across all diagnostic groups, between-group differences in ISI total score were modest, supporting a transdiagnostic perspective [4,5]. In multivariable analyses, a higher number of concurrent psychotropic medication classes was independently associated with clinically significant insomnia. However, in a sensitivity analysis additionally adjusting for benzodiazepine prescription, benzodiazepine prescription was associated with ISI ≥ 15, and the estimate for medication complexity was attenuated (Table S5). ADHD and depressive disorders were associated with higher odds compared with anxiety disorders. Due to the small size of the ADHD subgroup (*n* = 19) and the wide confidence intervals that this produces, findings related to ADHD should be interpreted cautiously and used to generate hypotheses. Eating disorders were excluded from the regression models due to the very small sample size (*n* = 4).

The overall prevalence of clinically significant insomnia in our sample is consistent with service-based studies that report a substantial proportion of psychiatric outpatients meet clinical thresholds when sleep is assessed systematically. These data represent a clinic-attending population rather than community prevalence; however, they may inform local workload planning and support estimates of potential demand for sleep-focused interventions in outpatient services [12,13].

Between-diagnosis differences in ISI total scores were statistically significant but of small effect size. This is consistent with a transdiagnostic interpretation: insomnia appears to be a shared clinical burden across psychiatric disorders. Additional diagnosis-linked variation likely reflects differences in symptom profiles, functional impairment, and illness course. In our cohort, depressive disorders showed higher ISI scores than anxiety disorders, which is consistent with clinical descriptions of insomnia as a core feature of depression, as well as with evidence that insomnia has been identified as a risk factor for depression and may act as a maintaining factor for depressive symptoms. At the same time, however, anxiety disorders still exhibited a clinically significant proportion of patients with ISI scores above 15, consistent with systematic reviews describing the complex bidirectional relationship between insomnia and anxiety [6,11,17,18,19,20,21].

Item- and domain-level findings suggest that between-diagnosis differences may be more apparent in perceived impact and distress than in nocturnal symptoms alone. Clinically, this matters because dissatisfaction, daytime interference and distress often drive help-seeking and may respond to psychoeducation and CBT-I components even when comorbid psychiatric symptoms persist. Chronobiological considerations may also be relevant, particularly in depressive disorders, where circadian dysregulation can influence sleep timing and early morning awakening and may affect treatment response [22]. Circadian factors may also be relevant in cases of bipolar disorder and ADHD, where irregular sleep–wake patterns and phase delay tendencies have been observed [19,23,24]. In ADHD, delayed sleep phase and stimulant-related effects are plausible contributors to insomnia complaints, but structured information on stimulant exposure and circadian phase was not available [23,24]. Personality disorders also showed a high proportion of clinically significant insomnia in our sample, in keeping with reports of chronic sleep disturbance and insomnia in borderline personality disorder [25,26].

Medication complexity was identified as a factor associated with clinically significant insomnia in the initial multivariable model. However, when the variable ‘prescription of benzodiazepines’ (yes/no) was added as an additional covariate, the association between benzodiazepine prescription and ISI ≥ 15 remained significant, whereas the estimate for medication complexity was attenuated (Table S5). This suggests that the class-count metric may partly capture hypnotic prescribing in response to sleep complaints and distress rather than indicating a direct effect of broader regimen complexity on insomnia severity. Confounding by indication and reverse causation are plausible, given that psychotropic polypharmacy often occurs alongside greater global illness severity and treatment resistance in routine psychiatric care [27,28]. It is important to note that these retrospective cross-sectional data do not allow us to determine directionality (i.e., whether a greater insomnia burden leads to more complex prescribing or whether more complex regimens contribute to insomnia symptoms). Benzodiazepine prescriptions were common among this outpatient sample (see Table S3). While information on dose, duration, timing and prescribing indication was not collected, the presence of clinically significant insomnia alongside benzodiazepine prescriptions highlights the importance of longitudinal monitoring and regular medication reviews. In routine psychiatric care, the relationship between insomnia complaints and benzodiazepine prescribing is likely bidirectional. Without longitudinal data and medication-level granularity, these mechanisms cannot be disentangled; therefore, these observational data do not support causal claims regarding benzodiazepines and insomnia severity [1,2,3,29]. Z-drug prescriptions were uncommon in this cohort (3.4%), which may reflect local prescribing practices and availability, and should not be generalized beyond this single-center setting. During the study period, newer hypnotics such as dual orexin receptor antagonists (e.g., daridorexant) and prescription melatonin receptor agonists were not part of routine clinical practice in our public community mental health setting, largely due to limited reimbursement within the public health system and high out-of-pocket costs. Accordingly, these agents were not prescribed and were not represented in our routine medication dataset.

From a service perspective, these findings could inform practical quality improvement initiatives, such as incorporating brief insomnia screening into routine appointments and documenting follow-up actions. Where resources allow, behavioral sleep interventions could be considered and evaluated prospectively. These interventions could include brief components of cognitive behavioral therapy for insomnia (CBT-I), group formats, digital CBT-I or referral pathways. However, access to formal CBT-I pathways may be limited in community psychiatry settings, so the feasibility of implementation should be assessed through dedicated, service-level studies with outcome monitoring (e.g., repeat Insomnia Severity Index (ISI) scores) [1,2,3,9,10]. Insomnia has also been linked to continued engagement with outpatient psychiatric services, highlighting the potential relevance of systematic sleep assessment at a service level [30].

### 5.1. Limitations

Several limitations should be considered. First, this was a single-center study within one Italian community mental health service. Care pathways, diagnostic mix and prescribing patterns may differ in other services and regions, which limits the generalizability of the findings. Second, as the study is retrospective and cross-sectional, causal inferences regarding diagnostic group differences or the relationship between medication complexity and insomnia cannot be made. Third, the recruitment period was three months in early autumn (September–November 2025), which may not have captured seasonal variations in sleep symptoms or service utilization. Repeating the study over a longer period spanning multiple seasons would improve generalizability. Fourth, our analyses relied on routinely collected variables and we lacked standardized measures of psychiatric symptom severity, duration of illness and detailed functional outcomes, which may have led to residual confounding factors. Fifth, medication variables were captured at the class level, without information on dose, timing or duration. Confounding by indication and reverse causation are likely, and associations between medications should not be interpreted as effects of individual drugs. In addition, the class-count approach may equate qualitatively different regimens (e.g., an antidepressant and a benzodiazepine versus multiple agents within the same class) and does not take into account polypharmacy within a class, clinical intent or dose-dependent effects. Moreover, benzodiazepines were prescribed to 51.8% of the sample and antipsychotics to 37.7%, but information on dose, duration, timing or prescribing indication was not provided. This is a significant uncontrolled factor as benzodiazepine exposure can influence subjective sleep complaints (e.g., acute sedation, tolerance, and withdrawal-related sleep disturbances), and may be more commonly prescribed to patients experiencing greater distress, which could distort ISI-based comparisons. Sixth, insomnia was assessed using a self-report measure referencing the previous two weeks. We did not systematically assess other sleep disorders, such as obstructive sleep apnoea, restless legs syndrome/periodic limb movement disorder and circadian rhythm sleep–wake disorders. We also did not assess substance use, shift work, caffeine and nicotine consumption, or psychosocial stressors, including socioeconomic burden and occupational instability. Objective sleep measures (e.g., actigraphy or polysomnography) were also not assessed. These factors may have contributed to residual confounding and unexplained variance. Furthermore, given this cohort’s substantial exposure to benzodiazepines and antipsychotics, the absence of systematic screening for sleep-disordered breathing and other sleep disorders means that ISI scores may reflect a range of sleep pathologies rather than insomnia specifically. Finally, some diagnostic groups were small (notably eating disorders and ADHD), leading to wider uncertainty, and these subgroup findings should be interpreted as hypothesis-generating.

### 5.2. Future Directions

Future work should extend these findings in larger multicenter designs with longer recruitment windows across seasons and prospective follow-up. Incorporating objective sleep measures and standardized psychiatric symptom severity scales would help disentangle insomnia-related distress from overall illness burden. Pragmatic implementation studies that evaluate feasible behavioral sleep interventions (including components of cognitive behavioral therapy for insomnia (CBT-I)), with prospective outcome monitoring in community psychiatric services, are needed.

## 6. Conclusions

In conclusion, clinically significant insomnia was common among psychiatric outpatients attending a community mental health center. A higher insomnia burden was observed in patients with depressive disorders, and medication complexity was associated with clinically significant insomnia. Integrating a structured assessment of insomnia into routine psychiatric care could help to identify patients who require further sleep-focused assessment and facilitate access to evidence-based interventions where these are available. Prospective studies are needed to clarify the clinical impact of this approach in routine services.

## Figures and Tables

**Table 1:** Demographic and clinical characteristics of the sample.

Variable	Value
Sample size	506
Age, mean (SD), years	45.1 (16.7)
Female sex, *n* (%)	265 (52.4)
Education, mean (SD), years	12.4 (3.6)
Psychiatric comorbidity, *n* (%)	170 (33.6)
On psychotropic treatment, *n* (%)	463 (91.5)
Medication complexity (psychotropic classes), mean (SD)	1.86 (1.03)
Antidepressants, *n* (%)	274 (54.2)
Antipsychotics, *n* (%)	191 (37.7)
Benzodiazepines, *n* (%)	262 (51.8)
Antiepileptics/mood stabilizers, *n* (%)	118 (23.3)
Lithium, *n* (%)	57 (11.3)
Z-drugs, *n* (%)	17 (3.4)
Other psychotropics, *n* (%)	21 (4.2)
ISI total score, mean (SD)	12.18 (6.99)
Clinically significant insomnia (ISI ≥ 15), *n* (%)	205 (40.5)
ISI severity: None, *n* (%)	144 (28.5)
ISI severity: Subthreshold, *n* (%)	157 (31.0)
ISI severity: Moderate, *n* (%)	150 (29.6)
ISI severity: Severe, *n* (%)	55 (10.9)
**Primary diagnosis (broad group)**	
Eating disorders	4 (0.8)
Anxiety disorders	84 (16.6)
Obsessive–compulsive disorder	31 (6.1)
Adjustment disorder	29 (5.7)
Psychotic disorders	52 (10.3)
Bipolar disorders	125 (24.7)
ADHD	19 (3.8)
Personality disorders	42 (8.3)
Depressive disorders	120 (23.7)

Notes: Data are presented as mean (SD) or *n* (%). Medication complexity indicates the number of concurrent psychotropic medication classes. Abbreviations: ADHD, attention-deficit/hyperactivity disorder; ISI, Insomnia Severity Index; OCD, obsessive–compulsive disorder; SD, standard deviation.

**Table 2:** ISI total score, domain scores, and prevalence of clinically significant insomnia (ISI ≥ 15) by diagnostic group.

Diagnostic Group	*n*	ISI Total, Mean (SD)	Nocturnal Domain *, Mean (SD)	Impact/Distress Domain †, Mean (SD)	ISI ≥ 15, *n* (%)
Anxiety disorders	84	10.17 (6.63)	4.04 (2.74)	6.13 (4.25)	24 (28.6)
Adjustment disorder	29	10.72 (7.25)	4.59 (2.96)	6.14 (4.55)	8 (27.6)
ADHD	19	13.00 (5.47)	4.68 (1.92)	8.32 (3.89)	11 (57.9)
Bipolar disorders	125	12.75 (6.84)	4.78 (2.83)	7.98 (4.30)	49 (39.2)
Depressive disorders	120	13.87 (7.09)	5.40 (2.83)	8.47 (4.51)	63 (52.5)
Obsessive–compulsive disorder	31	10.48 (6.66)	3.97 (2.64)	6.52 (4.23)	11 (35.5)
Personality disorders	42	13.31 (7.01)	5.00 (3.02)	8.31 (4.28)	21 (50.0)
Psychotic disorders	52	11.08 (7.18)	4.29 (2.78)	6.79 (4.75)	17 (32.7)
Eating disorders	4	8.00 (7.96)	3.00 (2.94)	5.00 (5.03)	1 (25.0)

Notes: * Nocturnal domain = sum of ISI items 1a–1c (sleep onset, sleep maintenance, early awakening). † Impact/distress domain = sum of ISI items 2–5. Abbreviations: ADHD, attention-deficit/hyperactivity disorder; ISI, Insomnia Severity Index; SD, standard deviation.

**Table 3:** Multivariable logistic regression for clinically significant insomnia (ISI ≥ 15).

Predictor	OR (95% CI)	*p*-Value
ADHD vs. Anxiety disorders	3.64 (1.26–10.55)	0.017
Adjustment disorder vs. Anxiety disorders	0.93 (0.36–2.45)	0.890
Bipolar disorders vs. Anxiety disorders	1.03 (0.54–2.00)	0.920
Depressive disorders vs. Anxiety disorders	1.99 (1.07–3.73)	0.031
Obsessive–compulsive disorder vs. Anxiety disorders	1.17 (0.48–2.87)	0.732
Personality disorders vs. Anxiety disorders	1.97 (0.86–4.52)	0.110
Psychotic disorders vs. Anxiety disorders	1.11 (0.51–2.43)	0.792
Age (per year)	1.01 (1.00–1.02)	0.225
Female sex	1.39 (0.95–2.03)	0.092
Education (per year)	1.02 (0.97–1.07)	0.490
Medication complexity (per additional class)	1.43 (1.16–1.77)	<0.001

Notes: Reference diagnostic group: anxiety disorders. Covariates: age, sex, years of education, and medication complexity (number of concurrent psychotropic medication classes). The eating disorders group was excluded from multivariable models because of the very small sample size (*n* = 4).

## Data Availability

De-identified participant data may be available from the corresponding author upon reasonable request, subject to institutional approvals and applicable data protection regulations.

## Supplementary Materials

The following supporting information can be downloaded at https://www.mdpi.com/article/10.3390/brainsci16060617/s1, Figure S1. STROBE-style study flow diagram summarizing patient selection. Table S1. ISI psychometric properties. Cronbach’s alpha: 0.924. Table S2. Association between diagnostic group and ISI severity categories. Table S3. Medication class prevalence by diagnostic group (percent). Table S4. Ordinal logistic regression for ISI severity categories (ordered outcome). Table S5. Sensitivity analysis for clinically significant insomnia (ISI ≥ 15) including benzodiazepine prescription.

## Abbreviations

ADHD, attention-deficit/hyperactivity disorder; ANOVA, analysis of variance; CBT-I, cognitive behavioral therapy for insomnia; CMHC, Community Mental Health Center; ISI, Insomnia Severity Index; OCD, obsessive–compulsive disorder; SCID-5, Structured Clinical Interview for DSM-5; SD, standard deviation.
