# Supplementary material for: Insomnia Severity in Psychiatric Outpatients: Real-World Insomnia Severity Index Data from an Italian Community Mental Health Center
Source: Brain Sci. 2026 Jun 9;16(6):617. doi: 10.3390/brainsci16060617 (PMC13297384; doi:10.3390/brainsci16060617)
Supplement: Supplementary file 1 [file brainsci-16-00617-s001.zip › brainsci-4326696-supplementary.pdf]

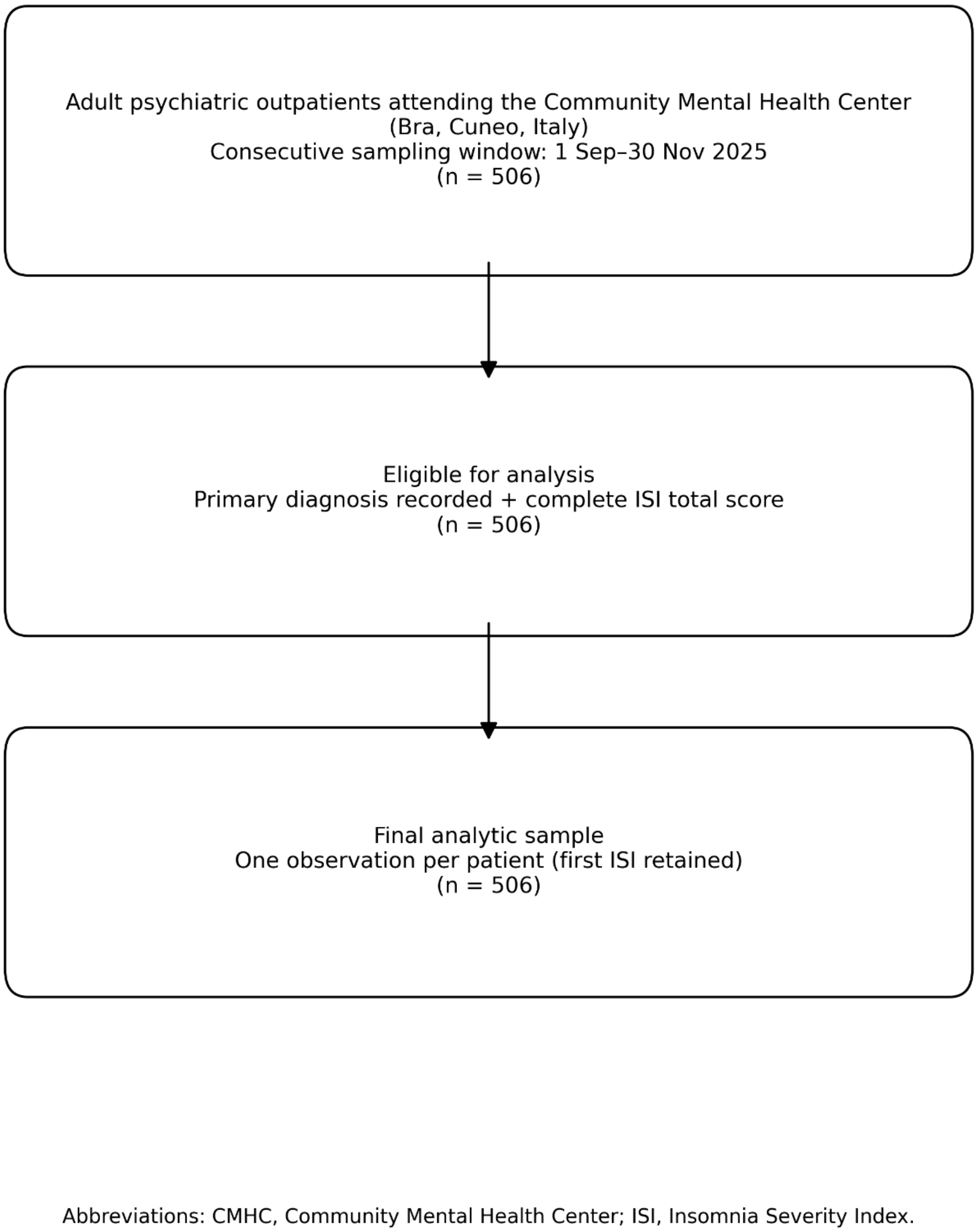

Figure S1. STROBE-style study flow diagram summarizing patient selection.

Abbreviations: ISI, Insomnia Severity Index

Table S1. ISI psychometric properties. Cronbach's alpha: 0.924.

ISI item	Item code	Corrected item–total r
Sleep onset	1.a	0.657
Sleep maintenance	1.b	0.807
Early awakening	1.c	0.605
Sleep satisfaction	2	0.876
Daily interference	3	0.795
Noticeability	4	0.727
Distress	5	0.861

Table S2. Association between diagnostic group and ISI severity categories.

Association	Chi-square	df	p	Cramer's V
Diagnosis × ISI severity (none/subthreshold/moderate/severe)	41.44	24	0.0149	0.165

Table S3. Medication class prevalence by diagnostic group (percent).

Diagnosis	Antidepressants (%)	Antipsychotics (%)	Benzodiazepines (%)	Antiepileptics mood stabilizers (%)	Lithium (%)	Z-drugs (%)	Other psychotropics (%)	Polytherapy (≥2 psychotropic medications) (%)
Eating disorders	50.0	25.0	25.0	0.0	0.0	0.0	0.0	25.0
Anxiety disorders	56.0	3.6	54.8	1.2	0.0	3.6	1.2	41.7
Obsessive-compulsive disorder	90.3	19.4	51.6	6.5	0.0	0.0	0.0	54.8
Adjustment disorder	41.4	6.9	44.8	3.4	0.0	6.9	0.0	20.7
Psychotic disorders	9.6	96.2	44.2	21.2	7.7	5.8	1.9	78.8
Bipolar disorders	43.2	66.4	47.2	47.2	38.4	3.2	3.2	86.4
ADHD	21.1	10.5	21.1	15.8	5.3	0.0	73.7	47.4
Personality disorders	42.9	40.5	57.1	64.3	9.5	0.0	0.0	71.4
Depressive disorders	86.7	22.5	63.3	11.7	0.0	4.2	0.8	72.5

Table S4. Ordinal logistic regression for ISI severity categories (ordered outcome).

**Predictor**	**OR (95% CI)**	**p-value**
ADHD vs Anxiety disorders	2.88 (1.19–6.95)	0.019
Adjustment disorder vs Anxiety disorders	0.93 (0.41–2.07)	0.850
Bipolar disorders vs Anxiety disorders	1.17 (0.67–2.03)	0.581
Depressive disorders vs Anxiety disorders	2.08 (1.21–3.57)	0.008
Obsessive-compulsive disorder vs Anxiety disorders	1.13 (0.51–2.47)	0.770
Personality disorders vs Anxiety disorders	1.86 (0.90–3.84)	0.092
Psychotic disorders vs Anxiety disorders	1.28 (0.67–2.45)	0.458
Age (per year)	1.01 (1.00–1.02)	0.017
Female sex	1.21 (0.87–1.69)	0.250
Education (per year)	1.01 (0.96–1.05)	0.753
Medication complexity (per additional class)	1.43 (1.20–1.72)	<0.001

Note: Reference diagnostic group: anxiety disorders. Covariates: age, sex, years of education, and medication complexity (number of concurrent psychotropic medication classes). The eating disorders group was excluded from multivariable models because of the very small sample size (n=4).

Table S5. Sensitivity analysis for clinically significant insomnia (ISI ≥ 15) including benzodiazepine prescription.

Predictor	OR (95% CI)	p-value
ADHD vs Anxiety disorders	4.79 (1.61–14.27)	0.005
Adjustment disorder vs Anxiety disorders	0.98 (0.37–2.57)	0.961
Bipolar disorders vs Anxiety disorders	1.42 (0.70–2.88)	0.332
Depressive disorders vs Anxiety disorders	2.23 (1.18–4.21)	0.014
Obsessive-compulsive disorder vs Anxiety disorders	1.32 (0.53–3.27)	0.553
Personality disorders vs Anxiety disorders	2.36 (1.01–5.51)	0.048
Psychotic disorders vs Anxiety disorders	1.35 (0.60–3.00)	0.468
Age (per year)	1.01 (0.99–1.02)	0.269
Female sex	1.38 (0.94–2.02)	0.101
Education (per year)	1.02 (0.96–1.07)	0.528
Medication complexity (per additional class)	1.17 (0.90–1.53)	0.233
Benzodiazepine prescription (yes vs no)	1.82 (1.13–2.95)	0.015

Note: Reference diagnostic group: anxiety disorders. Covariates: age, sex, years of education, medication complexity (number of concurrent psychotropic medication classes), and benzodiazepine prescription (yes/no). The eating disorders group was excluded from multivariable models because of the very small sample size (n=4).
